# Ultrasensitive SERS detection of trace malachite green using noble-metal-free EuFeO_3_/ZnO@Ti_3_C_2_T_*x*_ MXene heterostructures

**DOI:** 10.1039/d6ra04328c

**Published:** 2026-08-04

**Authors:** Thi Thanh Huong Nguyen, Anh Tien Nguyen, Tri Danh Giang, Thi Thuy Pham, Xuan Vuong Bui, Thi Lan Huong Nguyen, Huu Tri Nguyen, Ngoc Nhiem Dao, Thi Thanh Thuy Le

**Affiliations:** a Faculty of Environment and Labour Safety, Ton Duc Thang University 19 Nguyen Huu Tho Street, Tan Hung Ward Ho Chi Minh City 700000 Vietnam; b Faculty of Chemistry, Ho Chi Minh City University of Education Ho Chi Minh City 700000 Vietnam; c Faculty of Natural Sciences Education, Sai Gon University 273 An Duong Vuong Street, Cho Quan Ward Ho Chi Minh City 700000 Vietnam lttthuy@sgu.edu.vn; d Institute of Materials Science, Vietnam Academy of Science and Technology 18 Hoang Quoc Viet Street, Nghia Do Ward Hanoi 100000 Vietnam

## Abstract

Malachite green (MG), an illegally used aquaculture dye with carcinogenic and mutagenic effects, remains a persistent contaminant in aquatic environments and food chains, necessitating the development of highly sensitive and reliable analytical platforms for trace-level monitoring. In this study, a hierarchical EuFeO_3_/ZnO@Ti_3_C_2_T_*x*_ MXene heterostructure was successfully engineered as a noble-metal-free surface-enhanced Raman scattering (SERS) substrate for ultrasensitive MG detection in aquaculture water. The hierarchical assembly of EuFeO_3_ and ZnO on conductive Ti_3_C_2_T_*x*_ MXene generated electronically coupled heterointerfaces that facilitated rapid charge transfer and efficient carrier transport. Structural and spectroscopic analyses confirmed the successful formation of the ternary architecture with intimate interfacial integration, enhanced visible-light absorption, enlarged surface accessibility, and significantly suppressed charge-carrier recombination compared with pristine and binary systems. Consequently, the EuFeO_3_/ZnO@Ti_3_C_2_T_*x*_ substrate delivered markedly amplified Raman responses, achieving ultrasensitive MG detection at concentrations as low as 10^−9^ M with an enhancement factor of 7.31 × 10^6^. The substrate further demonstrated excellent linearity, reproducibility, and stable analytical performance in complex aquaculture water matrices, achieving recoveries ranging from 82.7% to 111.8% with relative standard deviations below 13.8%. Mechanistic investigations revealed that the enhanced SERS activity was predominantly governed by chemical enhancement arising from interfacial charge-transfer interactions between the heterostructure and MG molecules, while the conductive MXene scaffold accelerated electron transport and strengthened analyte-substrate coupling. This work not only demonstrates an effective strategy for constructing MXene-based semiconductor heterostructures with enhanced charge-transfer characteristics, but also provides a promising noble-metal-free SERS platform for practical food safety monitoring and environmental contaminant detection.

## Abstract 2

Malachite green (MG), an illegal aquaculture dye with carcinogenic and mutagenic effects, persists in aquatic environments and food chains, requiring sensitive analytical platforms for trace-level monitoring.

## Graphical abstract

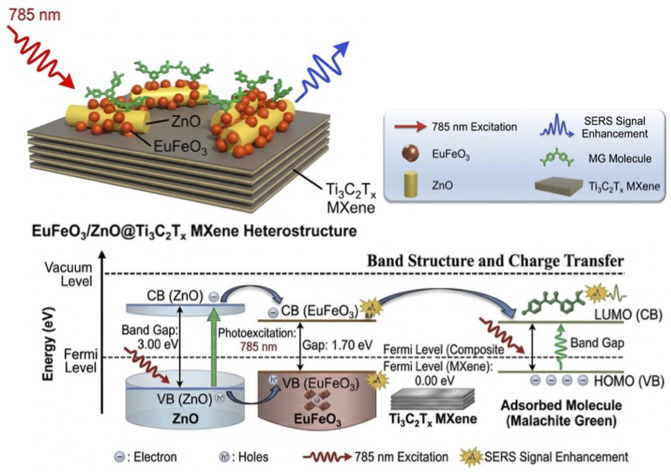

## 1. Introduction

Aquaculture has expanded rapidly over the past decades to meet the increasing global demand for affordable seafood and animal protein.^1^ However, intensive farming practices have also increased the vulnerability of aquaculture systems to chemical contamination originating from therapeutic agents, environmental pollutants, and illegal additives.^2–4^ Among these contaminants, synthetic dyes used as antifungal and antiparasitic agents have raised serious concerns due to their potential toxicity, persistence, and bioaccumulation behavior.^5^ In particular, malachite green (MG) has been historically applied for disease control in aquaculture due to its strong antimicrobial effectiveness; nevertheless, MG is strictly regulated or banned in many countries because of its mutagenic, carcinogenic, and teratogenic risks, as well as its conversion into leucomalachite green, a persistent metabolite that can accumulate in fish tissues.^6–8^ Consequently, the development of rapid, sensitive, and field-adaptable analytical methods for MG monitoring in fish pond water remains critical for aquaculture safety, environmental protection, and public health.^9,10^

Surface-enhanced Raman scattering (SERS) has been considered a promising analytical technique due to its molecular fingerprinting capability, rapid analysis, and high sensitivity, enabling detection of trace organic contaminants in complex matrices.^11,12^ Mechanistically, SERS enhancement originates mainly from two contributions: electromagnetic enhancement (EM) induced by localized surface plasmon resonance on metallic nanostructures, and chemical enhancement (CM) associated with interfacial charge transfer (CT) and modulation of molecular polarizability at the substrate-analyte interface.^13–15^ Therefore, the practical performance of SERS strongly depends on the development of substrates with high enhancement efficiency, stability, and reproducibility. Traditional noble-metal substrates (Au and Ag) provide strong EM enhancement; however, their high cost and susceptibility to oxidation often compromise long-term reliability under realistic aquatic conditions.^16–18^ Moreover, in complex aqueous matrices such as fish pond water, inconsistent analyte adsorption and non-uniform hotspot distribution may lead to poor signal reproducibility and reduced analytical confidence.^19^ These limitations motivate continued efforts toward robust and cost-effective SERS substrates for real-world aquaculture monitoring.

To address these challenges, noble-metal-free SERS substrates have received increasing attention as cost-effective and chemically stable alternatives. In recent years, a wide range of semiconductors, including metal oxides (*e.g.*, ZnO, TiO_2_, WO_3_, SnO_2_, Fe_2_O_3_, and CeO_2_), metal chalcogenides (*e.g.*, MoS_2_, WS_2_, CdS, and CuS), and emerging 2D semiconductors (*e.g.*, g-C_3_N_4_ and black phosphorus), have been explored as noble-metal-free SERS substrates.^20^ In general, the SERS enhancement is attributed to by CT-driven CM, where the efficiency of interfacial electron transfer and molecular adsorption plays a decisive role in determining signal intensity and stability.^21,22^ Therefore, rational engineering of semiconductor band structures, defect chemistry, and interfacial coupling is critical to achieving sensitive and reproducible SERS detection.^23^ Among these semiconductors, ZnO has attracted considerable interest due to its low cost, scalable synthesis, high physicochemical stability in aqueous media, and relatively low toxicity. ZnO is an n-type semiconductor with a wide band gap of 3.3 eV, and its abundant surface defect states can facilitate adsorption and promote interfacial CT processes that contribute to CM-driven SERS enhancement.^24^ However, pristine ZnO often exhibits limited SERS sensitivity and poor reproducibility, mainly due to inefficient charge separation and weak CT strength toward aromatic dye molecules.^25^ Consequently, constructing heterojunctions has been widely recognized as an effective approach to improve CT efficiency and suppress electron–hole recombination, thereby boosting semiconductor-based SERS performance.

Perovskite-type oxides are attractive heterojunction partners for semiconductor SERS substrates because their electronic structure can be tailored to promote interfacial charge separation and charge-transfer processes.^26^ EuFeO_3_, a representative perovskite ferrite, has drawn increasing attention due to its narrower band gap, approximately 1.94–2.39 eV and favorable redox-active Fe-based framework, which can introduce electronically active surface sites and facilitate interfacial electron exchange.^27,28^ When coupled with wide-band-gap ZnO, EuFeO_3_ can form an efficient EuFeO_3_/ZnO heterojunction with complementary band structures, enabling improved charge redistribution and suppressed electron–hole recombination.^29^ Such heterojunction-induced charge separation is expected to strengthen CT interactions at the substrate-analyte interface, thereby boosting chemical-enhancement-driven Raman amplification for aromatic dye molecules such as malachite green.^22,23^ Nevertheless, oxide-based heterostructures may still suffer from nanoparticle aggregation and limited carrier mobility, which can reduce the uniformity of active sites and compromise signal stability, highlighting the need for a conductive scaffold to stabilize hybrid architectures and accelerate electron transport.^30^

Meanwhile, two-dimensional (2D) materials have emerged as next-generation substrates for chemical sensing and Raman enhancement. In particular, Ti_3_C_2_T_*x*_ MXene, a representative member of the MXene family, has attracted extensive attention due to its high electrical conductivity, large surface area, rich surface terminations (–OH, –O, –F), and strong adsorption capability.^31,32^ These features enable MXene to serve as an effective platform for anchoring functional nanomaterials and promoting fast electron transfer processes. Importantly, MXene can contribute to signal enhancement and improve substrate stability by preventing severe nanoparticle agglomeration and providing abundant active interfacial sites.^33,34^ Recent advances in semiconductor-based SERS have increasingly focused on the rational design of MXene-derived heterostructures and electronically coupled semiconductor interfaces to improve the trace detection of environmental and biological contaminants. Zhao and co-workers^35^ constructed a dual-layer g-C_3_N_4_@Ti_3_C_2_T_*x*_ heterostructure *via* electrodeposition, where the intimate semiconductor-MXene interface promoted photoinduced charge transfer and enabled highly sensitive label-free SERS detection of stage-specific prostate cancer-derived exosomes. Concurrently, Chen *et al.*^36^ developed a flexible Ti_3_C_2_T_*x*_@Ag SERS platform using an *in situ* self-reduction strategy, demonstrating that the synergistic combination of MXene and plasmonic Ag nanoparticles significantly improved the sensitivity and reproducibility for trace viral detection. More recently, Xu *et al.*^37^ incorporated Ti_3_C_2_(OH)_2_ MXene into hybrid perovskite matrices to optimize interfacial energy-level alignment, thereby facilitating wavelength-dependent photoinduced charge transfer and enhancing the intrinsic chemical enhancement mechanism under near-infrared excitation. Despite these important advances, most reported systems are still based on binary heterostructures, where the limited number of heterointerfaces restricts charge-transfer pathways and interfacial carrier separation. Therefore, constructing semiconductor heterostructures on MXene may represent a promising strategy to obtain high-performance SERS substrates.

Accordingly, the objectives of this study were to synthesize EuFeO_3_/ZnO nanocomposite hybrids anchored on Ti_3_C_2_T_*x*_ MXene and to assess its physicochemical properties and SERS performance for sensitive and reliable detection of malachite green in aquaculture water. The novelty of this work may represent the first report on designing EuFeO_3_/ZnO nanocomposite hybrids anchored on Ti_3_C_2_T_*x*_ MXene as a hierarchical noble-metal-free SERS substrate, in which synergistic interfacial coupling between perovskite EuFeO_3_, semiconductor ZnO, and conductive MXene enables enhanced charge-transfer interactions and improved analyte adsorption, resulting in significantly improved sensitivity and reproducibility compared to conventional single and binary systems. It is hypothesized that integrating narrow-band-gap EuFeO_3_ with wide-band-gap ZnO on a conductive Ti_3_C_2_T_*x*_ MXene scaffold will promote charge separation and facilitate electron transport *via* heterojunction formation and MXene-mediated mechanisms. This synergistic interaction is expected to strengthen interfacial charge-transfer processes and analyte–substrate coupling, thereby leading to higher SERS sensitivity and reproducibility. The study provides a rational strategy for MXene-supported heterostructures in chemical-enhancement-driven SERS applications for environmental and food safety.

## 2. Materials and methods

### 2.1. Materials

Titanium aluminum carbide (Ti_3_AlC_2_, MAX phase, ≥98%), sodium fluoride (NaF, ≥99%), hydrochloric acid (HCl, 37%, ACS reagent), zinc nitrate hexahydrate (Zn(NO_3_)_2_·6H_2_O, ≥98%), hexamethylenetetramine (C_6_H_12_N_4_, HMTA, ≥99%), europium(iii) nitrate hexahydrate (Eu(NO_3_)_3_·6H_2_O, 99.9% trace metals basis), iron(iii) nitrate nonahydrate (Fe(NO_3_)_3_·9H_2_O, ≥98%), sodium hydroxide (NaOH, ≥98%), citric acid monohydrate (C_6_H_8_O_7_·H_2_O, ≥99.5%), ethanol (C_2_H_5_OH, absolute, ≥99.5%), and malachite green oxalate (C_23_H_25_ClN_2_·C_2_H_2_O_4_, ≥90%) were purchased from Sigma-Aldrich (USA) and used as received. Deionize water was used throughout all experiments. All chemicals were of analytical grade and used as received without further purification.

### 2.2. Synthesis of Ti_3_C_2_T_*x*_ MXene

Ti_3_C_2_T_*x*_ MXene was prepared *via* an alkali-assisted hydrothermal etching method using concentrated NaOH as the etching reagent. Briefly, Ti_3_AlC_2_ powder (300 mg) was added to 30 mL NaOH solution (27.5 M) and stirred at room temperature for 5 min to obtain a uniform black MAX phase dispersion. The mixture was then transferred into a 50 mL Teflon-lined stainless-steel autoclave and heated at 90 °C for 24 h. After the reaction, the alkaline product was washed thoroughly with deionized water and centrifuged at 3500 rpm for 5 min per cycle until the pH of the supernatant reached approximately 7. The resulting solid was collected and dried at 60 °C overnight to obtain Ti_3_C_2_T_*x*_ powder for subsequent experiments.^38^

### 2.3. Synthesis of *o*-EuFeO_3_ nanoparticles

The *o*-EuFeO_3_ nanoparticles were prepared *via* a simple co-precipitation method following the procedure reported by Nguyen Anh Tien *et al.* (2023).^39^ Briefly, an aqueous solution (50 mL) containing Eu(NO_3_)_3_ and Fe(NO_3_)_3_ (0.15 M each) was added into 400 mL of boiling deionized water (>95 °C) under continuous stirring and maintained for 10 min to ensure complete hydrolysis. After cooling to room temperature, 0.1 M NaOH was added dropwise to adjust the pH to 10, resulting in precipitation. The suspension was stirred for 60 min, allowed to settle, and the precipitate was collected by filtration, washed with distilled water and ethanol, and dried at ambient conditions. The obtained powder was ground and used as a precursor for *o*-EuFeO_3_ formation.

### 2.4. Synthesis of ZnO nanoparticles

The zinc precursor was synthesized *via* a sol–gel route using oxalic acid as the complexing agent.^40^ In brief, 100 mL of 0.6 M H_2_C_2_O_4_ was added to 100 mL of 0.4 M Zn(CH_3_COO)_2_·2H_2_O under stirring to form a homogeneous solution. This solution was then introduced into a solvent mixture consisting of 17 mL ethanol, 0.4 mL HNO_3_, and 1.6 mL distilled water under continuous stirring. The mixture was stirred for 2 h and aged for 48 h. The resulting precursor was collected, washed with deionized water and ethanol, dried at 100 °C overnight, and calcined at 450 °C for 3 h to obtain crystalline ZnO.

### 2.5. Synthesis of EuFeO_3_/ZnO@Ti_3_C_2_T_*x*_ nanocomposites

EuFeO_3_/ZnO@Ti_3_C_2_T_*x*_ nanocomposites were synthesized *via* a sol–gel method. Briefly, 100 mL of 0.6 M H_2_C_2_O_4_ was added dropwise to 100 mL of 0.4 M Zn(CH_3_COO)_2_·2H_2_O under continuous stirring to form a clear zinc precursor solution. Separately, a solvent mixture containing 17 mL ethanol, 0.4 mL HNO_3_, and 1.6 mL distilled water was prepared, into which 0.2 g of delaminated Ti_3_C_2_T_*x*_ MXene and 0.1 g of EuFeO_3_ nanoparticles were dispersed under stirring. The zinc precursor was then added dropwise to this suspension at room temperature, followed by stirring for 2 h to form a stable sol. The mixture was aged for 48 h to induce gelation, dried at 100 °C for 24 h, and calcined at 450 °C for 3 h to obtain EuFeO_3_/ZnO@Ti_3_C_2_T_*x*_ nanocomposites. The final product was ground into fine powder after cooling to room temperature.

For comparison, EuFeO_3_@Ti_3_C_2_T_*x*_, ZnO@Ti_3_C_2_T_*x*_, and EuFeO_3_/ZnO were prepared using the same procedure while omitting Zn(CH_3_COO)_2_·2H_2_O, EuFeO_3_, or Ti_3_C_2_T_*x*_, respectively. In all cases, the obtained gels were dried at 100 °C for 24 h and calcined at 450 °C for 3 h under the same conditions as the ternary EuFeO_3_/ZnO@Ti_3_C_2_T_*x*_ nanocomposite.

### 2.6. Characterizations

The crystal structure and phase composition of the synthesized materials were examined by X-ray diffraction (XRD) using Cu Kα radiation (*λ* = 1.5406 Å) on a Bruker D8 Advance diffractometer (Bruker, Germany). Functional groups and chemical bonding were analyzed by Fourier-transform infrared spectroscopy (FTIR) using a JASCO FT/IR-4600 spectrometer (JASCO, Japan). The morphological characteristics, including particle shape and surface texture, were investigated using scanning electron microscopy (SEM, Hitachi S-4800, Japan), while detailed nanostructure and interfacial features were further examined by transmission electron microscopy (TEM, JEOL JEM-2100, Japan). Elemental composition and spatial distribution were analyzed by energy-dispersive X-ray spectroscopy (EDX) coupled with a JEOL JSM-6490 microscope (JEOL, Japan). The specific surface area and porosity were determined by nitrogen adsorption–desorption measurements at 77 K using a NOVAtouch LX4 surface area analyzer (Quantachrome Instruments, USA), with the surface area calculated by the Brunauer–Emmett–Teller (BET) method and pore size distribution derived using the Barrett–Joyner–Halenda (BJH) model. Optical properties were evaluated by UV-visible diffuse reflectance spectroscopy (UV-Vis DRS) using a Shimadzu UV-2600 spectrophotometer (Shimadzu, Japan), and the band gap energies were estimated from Tauc plots. Photoluminescence (PL) spectra were recorded at room temperature using a FluoroMax+ spectrofluorometer (Horiba Scientific, USA) with an excitation wavelength of 325 nm to investigate the recombination and interfacial separation behavior of photogenerated charge carriers. Raman and surface-enhanced Raman scattering (SERS) spectra were collected using a Renishaw InVia confocal Raman microscope (Renishaw, UK) equipped with a 785 nm excitation laser and a 10× objective lens.

### 2.7. Preparation of standard solutions and fish pond water samples

A malachite green (MG) stock solution (10^−3^ M) was prepared by dissolving an appropriate amount of MG in deionized water. From this stock solution, a series of working standard solutions with concentrations ranging from 10^−3^ to 10^−8^ M were obtained by successive dilution using deionized water as the solvent. All solutions were freshly prepared prior to SERS analysis.

Fish pond water samples were collected and filtered through a membrane filter to remove suspended solids and particulate matter. The filtered water was subsequently spiked with defined volumes of the MG stock solution to obtain MG-contaminated fish pond water samples with concentrations in the range of 10^−5^ to 10^−8^ M. The spiked samples were gently mixed to ensure homogeneous distribution of MG before SERS measurements.

### 2.8. SERS measurement

The SERS performance of the synthesized EuFeO_3_/ZnO@Ti_3_C_2_T_*x*_ substrate was evaluated using malachite green as a model contaminant. The SERS substrate was prepared by dispersing the hybrid nanocomposite powder in ethanol under ultrasonication, followed by drop-casting the suspension onto clean silicon slides (approximately 1 × 1 cm). The coated silicon substrates were dried at room temperature to form a uniform. For SERS analysis, 10 µL of the MG standard solution or MG-spiked fish pond water sample was drop-cast onto the prepared substrate and allowed to dry naturally at ambient conditions. Raman and SERS spectra were recorded using a confocal Raman microscope with a 785 nm excitation laser, selected to minimize fluorescence interference while maintaining sufficient Raman intensity. The acquisition time was set to 15 s per scan, and the laser power was maintained at 6.8 mW to minimize potential thermal effects. To evaluate signal uniformity and reproducibility, spectra were collected from at least three randomly selected locations on each substrate. Control experiments were performed using EuFeO_3_, ZnO, EuFeO_3_/ZnO, and ZnO@Ti_3_C_2_T_*x*_ substrates prepared under identical conditions to clarify the contribution of the hybrid nanocomposite architecture to the observed SERS enhancement.

## 3. Results and discussion

### 3.1. XRD analysis

**Fig. 1:**
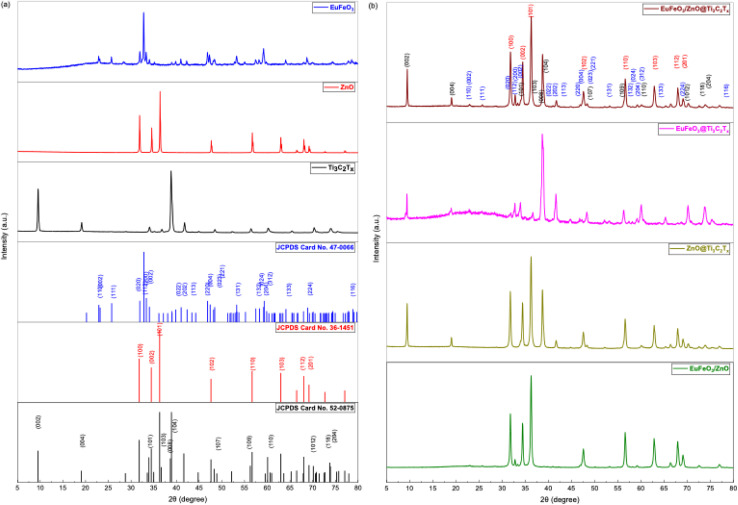
XRD patterns of (a) Ti_3_C_2_T_*x*_ MXene, ZnO, EuFeO_3_, and (b) their binary and ternary composites.

Fig. 1 shows the XRD patterns of the Ti_3_C_2_T_*x*_ MXene, ZnO, EuFeO_3_, and their corresponding binary and ternary composites. The XRD pattern of Ti_3_C_2_T_*x*_ MXene exhibits a series of diffraction peaks located at 2*θ* ≈ 9.5°, 19.1°, 34.1°, 38.8°, 41.8°, 48.8°, 56.4°, and 60.2°, which can be indexed to the (002), (004), (101), (104), (105), (107), (110), and (112) crystal planes, respectively. These reflections are characteristic of the layered Ti_3_C_2_T_*x*_ structure formed after selective etching of the Al layers from the Ti_3_AlC_2_ MAX phase.^41,42^ The prominent reflection observed at 2*θ* ≈ 38.8°, assigned to the (104) plane, confirms the structural transformation from the parent MAX phase and indicates successful Al removal during the etching process (Fig. S1). The presence of multiple non-basal reflections further suggests that the MXene retains a certain degree of long-range crystallographic order after etching, rather than forming a fully amorphous structure.

Following the identification of the Ti_3_C_2_T_*x*_ MXene phase, the XRD patterns of ZnO, EuFeO_3_/ZnO, ZnO@Ti_3_C_2_T_*x*_, and EuFeO_3_/ZnO@Ti_3_C_2_T_*x*_ nanocomposites exhibit a series of distinct diffraction peaks characteristic of the hexagonal wurtzite structure of ZnO. The prominent reflections located at 2*θ* ≈ 31.7°, 34.4°, 36.2°, 47.5°, 56.6°, 62.8°, and 68.0° can be indexed to the (100), (002), (101), (102), (110), (103), and (112) crystallographic planes, respectively. The calculated lattice parameters (*a* = 3.24982 Å and *c* = 5.20661 Å) are in good agreement with the standard values reported for hexagonal ZnO (JCPDS card no. 36-1451), confirming the formation of phase-pure ZnO with preserved crystal symmetry.^40,43^ Importantly, no additional diffraction peaks associated with zinc hydroxide, zinc carbonate, or other zinc-containing secondary phases are detected, indicating that the ZnO phase remains structurally stable during composite fabrication.

The XRD profiles of EuFeO_3_, EuFeO_3_/ZnO, EuFeO_3_@Ti_3_C_2_T_*x*_, and EuFeO_3_/ZnO@Ti_3_C_2_T_*x*_ exhibit a series of prominent diffraction peaks at 2*θ* ≈ 22.9° (101), 32.1° (121), 33.0° (040), 40.0° (202), 46.0° (242), 52.0° (123), 57.4° (204), and 67.2° (044), which can be well indexed to the orthorhombic perovskite structure of EuFeO_3_ (JCPDS card no. 47-0066). These diffraction features are in good agreement with previously reported studies on *o*-EuFeO_3_, confirming the structural integrity and phase stability of the synthesized EuFeO_3_.^39^ Furthermore, no diffraction peaks corresponding to common iron oxide impurities (such as α-Fe_2_O_3_ or Fe_3_O_4_) or europium oxide are detected, indicating the successful formation of phase-pure EuFeO_3_. The presence of multiple sharp and well-resolved reflections further confirms the good crystallinity of the EuFeO_3_ phase, which is essential for preserving its intrinsic electronic structure and Fe–O–Fe redox framework. In the EuFeO_3_/ZnO, EuFeO_3_@Ti_3_C_2_T_*x*_, and EuFeO_3_/ZnO@Ti_3_C_2_T_*x*_ composites, the characteristic EuFeO_3_ diffraction peaks are retained, although with slightly reduced intensity compared to pristine EuFeO_3_. This behavior suggests strong interfacial interactions between EuFeO_3_, ZnO, and the MXene support, while the absence of noticeable peak shifts indicates that the EuFeO_3_ crystal lattice remains intact. The incorporation of both ZnO and EuFeO_3_ onto the Ti_3_C_2_T_*x*_ MXene scaffold is therefore corroborated by the XRD results, demonstrating the robustness of the synthesis methodology and the structural stability of the resulting hybrid materials.

The XRD patterns of the EuFeO_3_/ZnO@Ti_3_C_2_T_*x*_ nanocomposites confirm the successful incorporation of ZnO, EuFeO_3_, and Ti_3_C_2_T_*x*_ MXene into a single composite architecture, as evidenced by the simultaneous presence of characteristic diffraction peaks corresponding to each individual phase. The preservation of the crystal structures of ZnO and EuFeO_3_, together with the retained MXene reflections, indicates that the composite formation occurs through interfacial assembly rather than bulk lattice substitution.

To gain further insight into the microstructural characteristics of the synthesized materials, the average crystallite size (*D*) was evaluated using the Debye–Scherrer equation (eqn (1)):^44^1
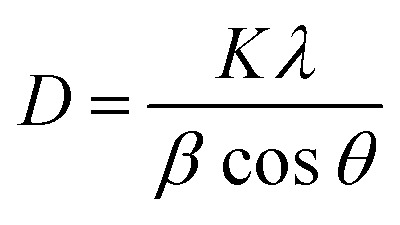
where *D*: average crystallite size (nm), *K*: Scherrer constant (a typical value is 0.94), *λ*: X-ray wavelength, *β*: the full width at half maximum (FWHM). Accordingly, the calculated crystallite sizes were 17.46 nm for ZnO, 19.8 nm for EuFeO_3_, 18.9 nm for EuFeO_3_/ZnO, 14.8 nm for ZnO@Ti_3_C_2_T_*x*_, 19.2 nm for EuFeO_3_@Ti_3_C_2_T_*x*_, and 16.3 nm for EuFeO_3_/ZnO@Ti_3_C_2_T_*x*_, respectively.

### 3.2. FT-IR spectrum

**Fig. 2:**
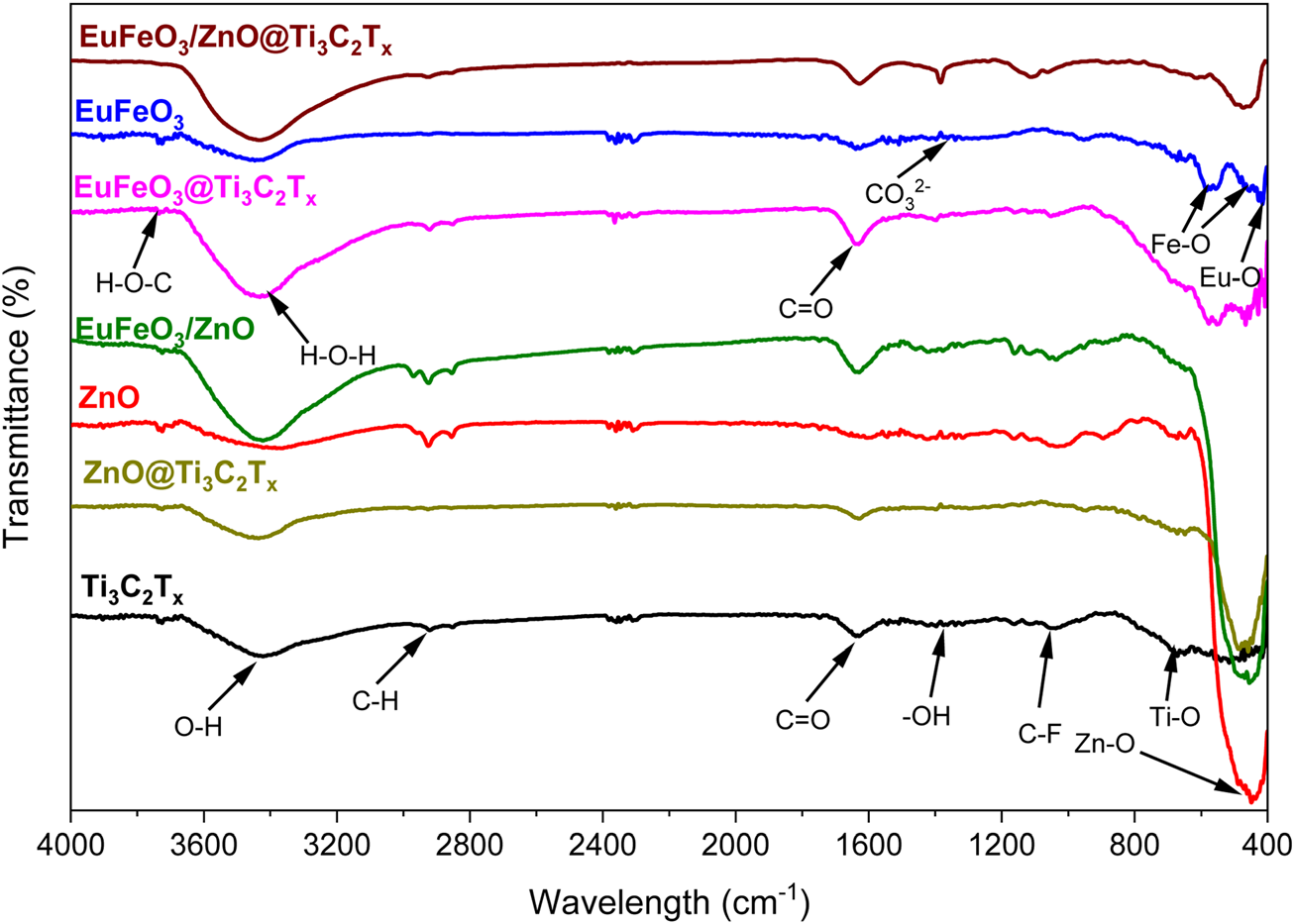
FT-IR spectra of Ti_3_C_2_T_*x*_ MXene, ZnO, EuFeO_3_, EuFeO_3_/ZnO, ZnO@Ti_3_C_2_T_*x*_, EuFeO_3_@Ti_3_C_2_T_*x*_, and EuFeO_3_/ZnO@Ti_3_C_2_T_*x*_ composites.

Fig. 2 reports the FT-IR spectra of Ti_3_C_2_T_*x*_ MXene, ZnO, EuFeO_3_, and their corresponding binary and ternary composites, providing further evidence of surface functional groups and interfacial interactions within the hybrid systems. In the EuFeO_3_/ZnO@Ti_3_C_2_T_*x*_ nanocomposite, the broad absorption band centered at approximately 3436 cm^−1^ and 1384 cm^−1^ are assigned to the stretching vibration of surface O–H groups, originating from hydroxyl terminations and adsorbed water molecules. The band observed near 1625 cm^−1^ is associated with C=O-related vibrations, indicating the presence of surface-adsorbed species. Additional absorption features at around 1217 cm^−1^ and 1047 cm^−1^ can be attributed to C–O and C–F stretching vibrations, respectively, which arise from the surface termination groups (–T_*x*_) of the MXene. The low-wavenumber band located at approximately 677 cm^−1^ corresponds to Ti–O vibrations, confirming the retention of the Ti_3_C_2_T_*x*_ framework within the composite.^45–49^

The characteristic absorption band appearing at around 452 cm^−1^ is assigned to Zn–O stretching vibrations, providing clear evidence for the presence of ZnO in the ternary composite. Furthermore, a weak absorption band observed at approximately 1403 cm^−1^ can be attributed to the symmetric stretching vibration of carbonate (CO_3_^2−^) groups, which is commonly associated with the formation of europium oxycarbonate (Eu_2_O_2_CO_3_) due to the adsorption of atmospheric CO_2_.^50^ Such carbonate-related features are frequently reported for rare-earth-based perovskite orthoferrites (RFeO_3_, where R = Y, La, Ho, Eu) and are generally considered surface-related rather than intrinsic lattice species.^39,50–53^ In addition, the absorption band detected at approximately 581 cm^−1^ can be assigned to the stretching vibration of Fe–O bonds within the FeO_6_ octahedra of europium orthoferrite, confirming the presence of the EuFeO_3_ phase.^39,51^ The band observed at around 475 cm^−1^ is associated with the bending vibration of O–Fe–O linkages, while the O–Eu–O bending modes characteristic of europium orthoferrite are recorded in the lower wavenumber region, typically in the range of 415 cm^−1^.^50^

Therefore, the FT-IR spectra of the binary and ternary composites retain the characteristic vibrational features of Ti_3_C_2_T_*x*_ MXene, ZnO, and EuFeO_3_, indicating that the individual components preserve their intrinsic structural motifs upon hybridization. The coexistence of MXene surface terminations together with Zn–O, Fe–O, and Eu–O vibrational modes provides strong evidence for the successful assembly of the composite materials through interfacial interactions, without the formation of new undesired phases.

### 3.3. EDX, SEM and TEM

**Fig. 3:**
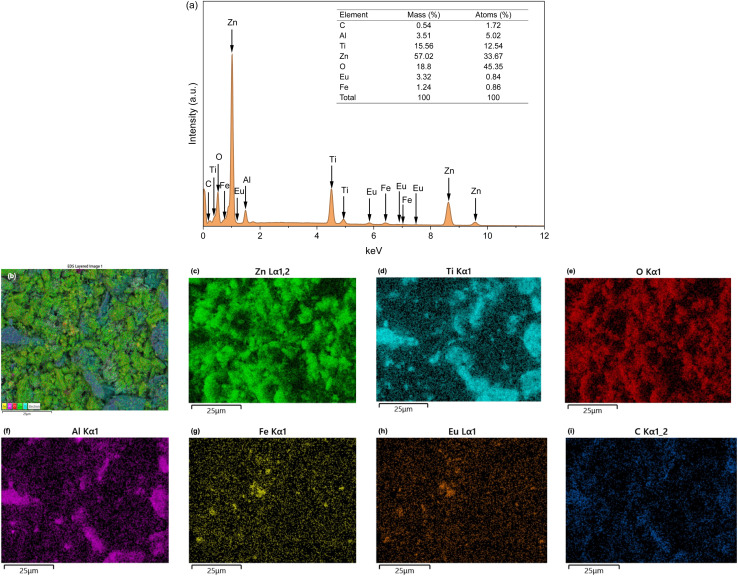
(a) EDX spectra and corresponding elemental composition tables of EuFeO_3_/ZnO@Ti_3_C_2_T_*x*_ MXene; (b–i) elemental mapping images of EuFeO_3_/ZnO@Ti_3_C_2_T_*x*_ MXene.

Fig. 3 presents the morphology and elemental analysis of the synthesized EuFeO_3_/ZnO@ Ti_3_C_2_T_*x*_ MXene heterostructure. The EDX spectrum (Fig. 3a) confirms the presence of Ti, C, O, Zn, Eu, and Fe, corresponding to the Ti_3_C_2_T_*x*_ MXene support, ZnO phase, and EuFeO_3_ component, respectively. In addition, a weak Al signal is detected, which can be attributed to trace residual Al species derived from the Ti_3_AlC_2_ MAX precursor after the etching process. Such residual Al is commonly observed in Ti_3_C_2_T_*x*_ MXene materials. To confirm the successful formation of heterojunction interfaces rather than simple, isolated mechanical blending, the nanoscale spatial distribution was evaluated using EDX elemental mapping (Fig. 3b–i). The element profiles for Zn (ZnO) and both Eu and Fe (EuFeO_3_) overlap across the exact same spatial coordinates on the Ti_3_C_2_T_*x*_ MXene sheets, with no phase segregation or isolated single-component domains detected. This nanoscale co-localization provides strong evidence of uniform interfacial contact between the two oxide phases. This intimate integration is a direct result of our *in situ* sol–gel synthesis methodology, where the ZnO precursor was chemically hydrolyzed and condensed directly onto the surfaces of pre-dispersed EuFeO_3_ nanoparticles and functionalized MXene templates. This approach creates highly interconnected, electronically coupled EuFeO_3_/ZnO heterointerfaces that are essential for the accelerated charge-carrier transport observed in our spectroscopic analyses.

**Fig. 4:**
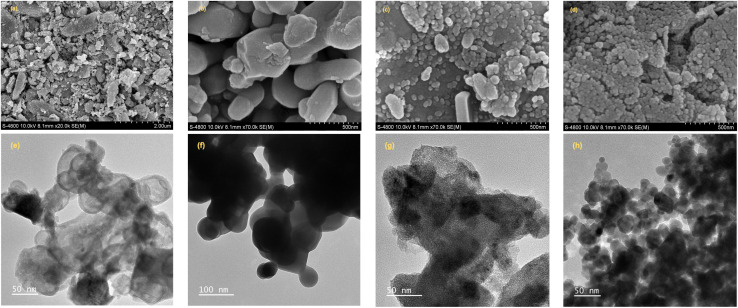
(a–d) SEM images of (a) Ti_3_C_2_T_*x*_ MXene, (b) EuFeO_3_, (c) ZnO@Ti_3_C_2_T_*x*_ MXene, (d) EuFeO_3_/ZnO@Ti_3_C_2_T_*x*_ MXene; (e–h) TEM images of (e) Ti_3_C_2_T_*x*_ MXene, (f) EuFeO_3_, (g) ZnO@Ti_3_C_2_T_*x*_ MXene, (h) EuFeO_3_/ZnO@Ti_3_C_2_T_*x*_ MXene.

Fig. 4a–d presents the SEM micrographs of Ti_3_C_2_T_*x*_ MXene, EuFeO_3_, ZnO@Ti_3_C_2_T_*x*_ MXene, and EuFeO_3_/ZnO@Ti_3_C_2_T_*x*_ MXene, respectively. The pristine Ti_3_C_2_T_*x*_ MXene (Fig. 4a) exhibits a characteristic accordion-like lamellar morphology composed of wrinkled and partially restacked nanosheets, confirming the effective exfoliation of the parent Ti_3_AlC_2_ MAX phase into a two-dimensional layered architecture. Such a morphology is highly favorable for subsequent surface functionalization because it provides a large exposed surface area and abundant nucleation sites for nanoparticle immobilization. The EuFeO_3_ sample (Fig. 4b) consists of nanosized particles with irregular to quasi-spherical geometry, forming aggregated clusters due to the intrinsically high surface energy of fine perovskite particles. After ZnO growth, the ZnO@Ti_3_C_2_T_*x*_ MXene composite (Fig. 4c) displays a dense distribution of nanoparticles anchored on the MXene sheets, resulting in an evidently roughened surface texture and indicating successful deposition of ZnO onto the conductive substrate. In the final EuFeO_3_/ZnO@Ti_3_C_2_T_*x*_ MXene heterostructure (Fig. 4d), oxide nanoparticles are homogeneously dispersed over the layered MXene framework, generating a compact and interconnected hierarchical architecture without severe agglomeration. This observation suggests that the MXene support effectively regulates particle dispersion during the multistep assembly process.

The TEM images shown in Fig. 4e–h further corroborate the structural features observed by SEM. The Ti_3_C_2_T_*x*_ MXene sample (Fig. 4e) retains its ultrathin and transparent nanosheet morphology, which is characteristic of successfully exfoliated two-dimensional materials. EuFeO_3_ (Fig. 4f) appears as nanoscale particles with higher contrast and partial aggregation, consistent with the presence of dense oxide domains. The ZnO@Ti_3_C_2_T_*x*_ MXene sample (Fig. 4g) confirms the intimate attachment of nanoparticles onto the MXene surface, demonstrating effective interfacial coupling between ZnO and the nanosheet support. As shown in Fig. 4h, the final composite exhibits close nanoscale integration among EuFeO_3_, ZnO, and Ti_3_C_2_T_*x*_ components, producing abundant heterointerfaces throughout the hybrid matrix. Such a well-organized multicomponent architecture is particularly advantageous for SERS applications, as the increased surface roughness, uniform distribution of active domains, and efficient interfacial charge-transfer pathways can collectively promote analyte adsorption and amplify Raman scattering signals, thereby enhancing detection sensitivity and signal reproducibility.

### 3.4. Surface area and porosity characteristics

**Fig. 5:**
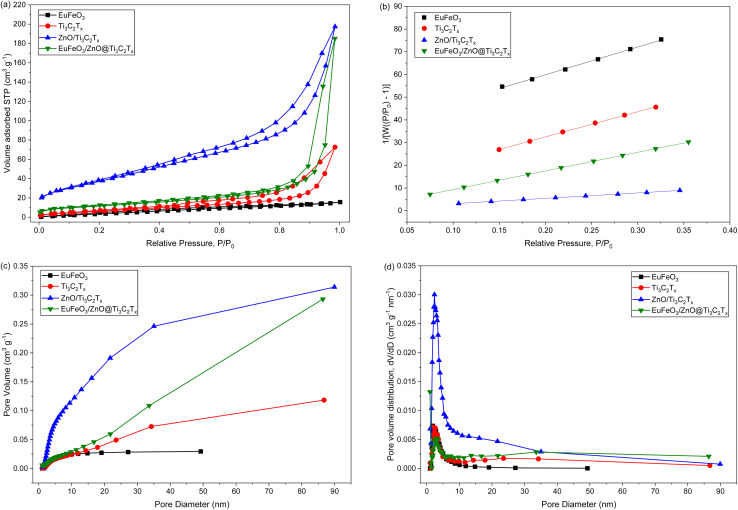
BET surface analysis based on N_2_ adsorption–desorption analysis of EuFeO_3_, Ti_3_C_2_T_*x*_ MXene, ZnO@Ti_3_C_2_T_*x*_, and EuFeO_3_/ZnO@Ti_3_C_2_T_*x*_: (a) adsorption–desorption isotherms, (b) BET specific surface area, (c) BJH pore size distribution, and (d) differential pore volume distribution.

The N2 adsorption–desorption analysis presented in Fig. 5 provides insight into the surface area and pore structure of EuFeO_3_, Ti_3_C_2_T_*x*_ MXene, ZnO@Ti_3_C_2_T_*x*_, and EuFeO_3_/ZnO@Ti_3_C_2_T_*x*_. As shown in Fig. 5a, all samples exhibit type IV adsorption–desorption isotherms with H3 hysteresis loops according to the IUPAC classification, confirming the mesoporous nature of the synthesized materials.^43^ This behavior is characteristic of layered solids or aggregates of plate-like particles that generate slit-shaped pores, which is consistent with the lamellar structure of Ti_3_C_2_T_*x*_ MXene and the interparticle voids formed after deposition of EuFeO_3_ and ZnO nanoparticles. All samples show gradual N_2_ uptake at low relative pressures followed by a pronounced increase at higher *P*/*P*_0_ values, indicating the coexistence of mesopores and larger textural voids. Among them, ZnO@Ti_3_C_2_T_*x*_ exhibits the highest adsorption capacity, suggesting that ZnO nanoparticles effectively suppress MXene restacking and create additional accessible pore channels. In contrast, pristine EuFeO_3_ shows the lowest uptake due to particle agglomeration and its compact structure, while the ternary EuFeO_3_/ZnO@Ti_3_C_2_T_*x*_ composite retains substantially enhanced adsorption behavior, confirming the formation of an open porous framework.

The BET specific surface area values shown in Fig. 5b and summarized in Table 1 further support these observations. EuFeO_3_ possesses the lowest surface area (31.034 m^2^ g^−1^), whereas Ti_3_C_2_T_*x*_ MXene exhibits a higher value (65.320 m^2^ g^−1^) due to its two-dimensional layered structure. A significant increase to 166.365 m^2^ g^−1^ is observed for ZnO@Ti_3_C_2_T_*x*_, indicating that ZnO nanoparticles act as spacers between MXene sheets, thereby preventing structural collapse and exposing additional active surface sites. After incorporation of EuFeO_3_, the surface area decreases to 66.473 m^2^ g^−1^ for EuFeO_3_/ZnO@Ti_3_C_2_T_*x*_, which can be attributed to partial pore occupation and surface coverage by EuFeO_3_ nanoparticles. Nevertheless, this value remains markedly higher than that of pristine EuFeO_3_, demonstrating that the composite retains a relatively accessible surface after hybridization.

**Table 1:** BET specific surface area, BJH pore diameter, and pore volume of the synthesized samples

	*S*_BET_ (m^2^ g^−1^)	Pore volume (cm^3^ g^−1^)	Pore diameter (nm)
EuFeO_3_	31.034	0.029	1.912
Ti_3_C_2_T_*x*_	65.32	0.118	1.897
ZnO@Ti_3_C_2_T_*x*_	166.365	0.314	2.379
EuFeO_3_/ZnO@Ti_3_C_2_T_*x*_	66.473	0.293	0.999

The pore structure analysis shown in Fig. 5c and d further confirms the hierarchical porosity of the synthesized materials. ZnO@Ti_3_C_2_T_*x*_ exhibits the highest cumulative pore volume (0.314 cm^3^ g^−1^) together with a broad pore size distribution, indicating the formation of abundant interconnected channels after ZnO deposition. In comparison, the EuFeO_3_/ZnO@Ti_3_C_2_T_*x*_ composite retains a relatively high pore volume (0.293 cm^3^ g^−1^) but shows a narrower and more refined pore size distribution, suggesting that EuFeO_3_ partially occupies or subdivides larger pores into smaller porous domains. This structural evolution leads to a more compact yet still accessible porous framework. These results demonstrate that the integration of ZnO and EuFeO_3_ onto Ti_3_C_2_T_*x*_ MXene effectively tailors the surface area and pore structure, resulting in a hierarchical porous system with preserved accessibility and structural stability.

### 3.5. Optical properties

**Fig. 6:**
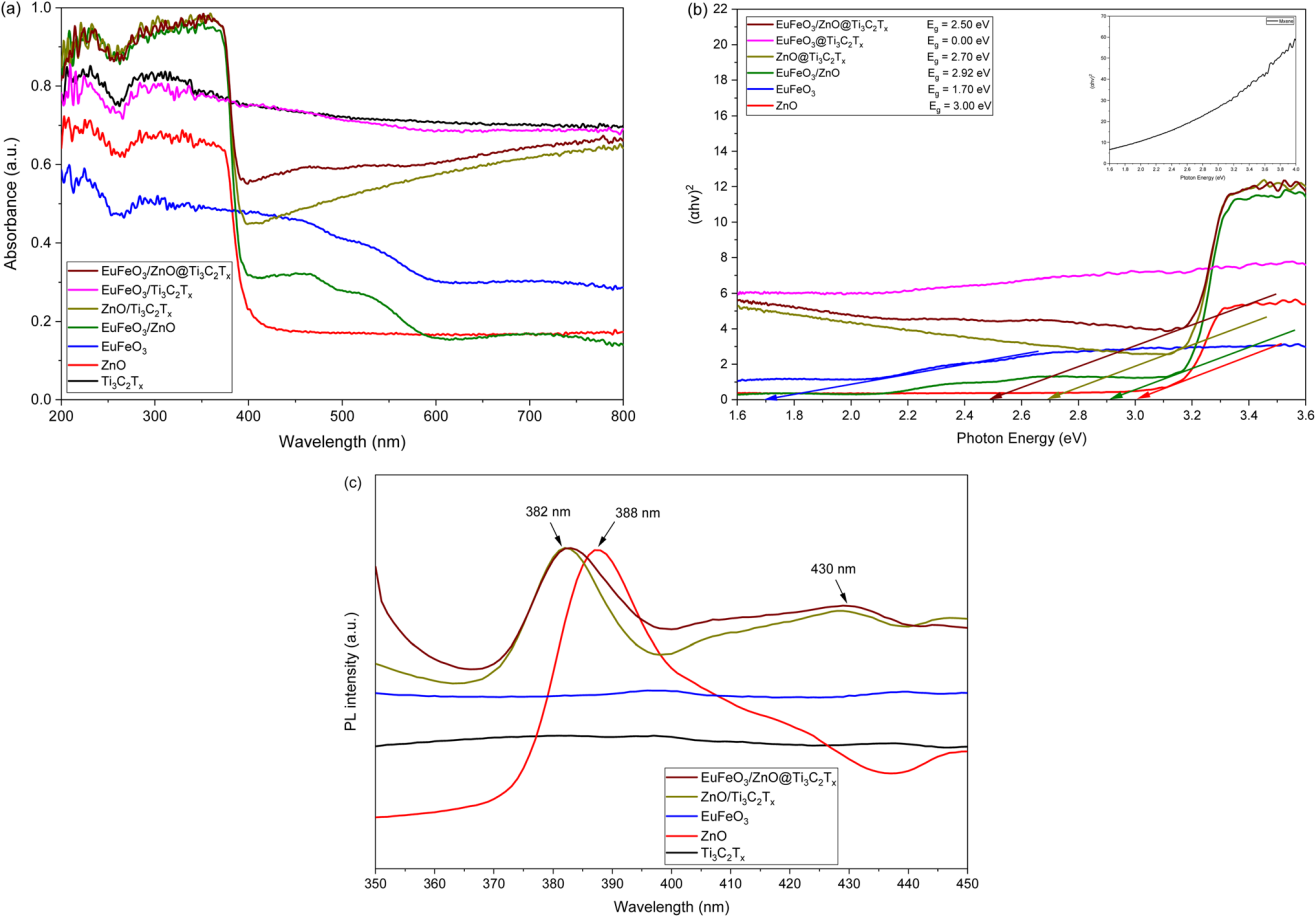
(a) UV-Vis DRS spectrum, (b) Tauc plot of Ti_3_C_2_T_*x*_ MXene, ZnO, EuFeO_3_, EuFeO_3_/ZnO, ZnO@Ti_3_C_2_T_*x*_, EuFeO_3_@Ti_3_C_2_T_*x*_, and EuFeO_3_/ZnO@Ti_3_C_2_T_*x*_, and (c) photoluminescence (PL) spectra of Ti_3_C_2_T_*x*_ MXene, ZnO, EuFeO_3_, EuFeO_3_/ZnO, and EuFeO_3_/ZnO@Ti_3_C_2_T_*x*_ recorded under an excitation wavelength of 325 nm (*λ*_e*x*_ = 325 nm).

As presented in Fig. 6a, pristine ZnO exhibits a sharp absorption edge in the UV region, with strong absorption below approximately 380–390 nm, which is characteristic of wide-band-gap semiconductors.^54^ In contrast, EuFeO_3_ shows a broader absorption profile extending well into the visible region, reflecting its narrower band gap and enhanced visible-light response. The Ti_3_C_2_T_*x*_ MXene displays broadband absorption across the entire UV-visible range without a distinct absorption edge, which can be attributed to its metallic-like conductivity and layered structure.^55^ For the binary EuFeO_3_/ZnO composite, the absorption intensity in the visible region is noticeably enhanced compared with pure ZnO, indicating that coupling ZnO with EuFeO_3_ effectively broadens the light-harvesting range.^56^ Upon further integration with MXene, the hybrid materials (ZnO@Ti_3_C_2_T_*x*_, EuFeO_3_@Ti_3_C_2_T_*x*_, and EuFeO_3_/ZnO@Ti_3_C_2_T_*x*_) exhibit significantly increased absorption throughout the visible region. In particular, the EuFeO_3_/ZnO@Ti_3_C_2_T_*x*_ heterostructure shows the highest and most continuous absorption intensity from the UV to visible range, which can be attributed to the synergistic contribution of EuFeO_3_ visible-light absorption, ZnO band-edge absorption, and the broadband light absorption of conductive MXene sheets.^57–59^ Such enhanced optical absorption is favorable for photocatalytic applications under visible-light irradiation.

In Fig. 6b, the optical band-gap energies (*E*_g_) of the pristine materials and hybrid materials were estimated from Tauc plots derived from the UV-Vis DRS data, following the relation (*αhν*) = *A*(*hν* − *E*_g_)^*n*/2^, where *α* is the absorption coefficient, *hν* is the photon energy, and *A* is a constant.^43^ The results reveal the following energy band gaps (*E*_g_) for the materials: 3.00 eV for ZnO, 1.70 eV for EuFeO_3_, 2.90 eV for EuFeO_3_/ZnO, 2.70 eV for ZnO@Ti_3_C_2_T_*x*_, 2.00 eV for EuFeO_3_@Ti_3_C_2_T_*x*_, and 2.50 eV for EuFeO_3_/ZnO@Ti_3_C_2_T_*x*_. Pristine ZnO exhibits a wide band gap, limiting its response to the UV region,^54^ whereas EuFeO_3_ shows a narrower band gap with enhanced visible-light absorption.^60^ EuFeO_3_ functions as a narrow-band-gap component that introduces additional electronic states and promotes interfacial charge transfer. The formation of the EuFeO_3_/ZnO heterojunction enables effective spatial separation of charge carriers, while the dispersion and interfacial contact of EuFeO_3_ on ZnO govern the density of active sites and charge-transfer efficiency relevant to SERS. The observed intermediate band gap of EuFeO_3_/ZnO further indicates electronic interaction between the two oxides, supporting the formation of a heterojunction rather than a solid-solution structure. Upon integration with Ti_3_C_2_T_*x*_ MXene, a further reduction in the apparent band gap is observed, which can be attributed to strong interfacial coupling and the introduction of additional electronic states near the band edges. As a result, optical transitions occur at lower photon energies, enhancing visible-light absorption.^61^ The ternary EuFeO_3_/ZnO@Ti_3_C_2_T_*x*_ exhibits the lowest apparent band gap, reflecting a synergistic effect of the combined components. This reduction is more likely associated with interfacial electronic interactions and charge-transfer processes rather than intrinsic band structure modification.

Photoluminescence (PL) analysis was conducted to investigate the recombination behavior of photogenerated electron–hole pairs and their implications for interfacial charge-transfer processes.^62^ As shown in Fig. 6c, all samples exhibit characteristic emission features in the UV-visible region, with prominent peaks at 382–388 nm corresponding to near-band-edge (NBE) excitonic recombination of ZnO, and a broader emission band centered around 430 nm attributed to defect-related states such as oxygen vacancies and surface trap levels.^63^ Lower PL intensity corresponds to suppressed radiative recombination and enhanced charge separation, whereas higher intensity reflects rapid recombination of charge carriers.^64^ Pristine ZnO displays the highest PL intensity, indicating fast electron–hole recombination. In contrast, the incorporation of EuFeO_3_ and Ti_3_C_2_T_*x*_ leads to a pronounced quenching of PL intensity, with the EuFeO_3_/ZnO@Ti_3_C_2_T_*x*_ composite exhibiting the lowest emission among all samples, evidencing more efficient charge separation and prolonged carrier lifetimes. This behavior can be attributed to the formation of a heterojunction interface between ZnO and EuFeO_3_, together with defect-related energy levels (*e.g.*, oxygen vacancies) that facilitate interfacial charge transfer and suppress electron–hole recombination.^65,66^ Moreover, the highly conductive Ti_3_C_2_T_*x*_ MXene acts as an efficient electron sink, enabling rapid electron extraction and transport, thereby further enhancing charge separation.^67^ In addition, the structural characteristics of Ti_3_C_2_T_*x*_ MXene, such as layer thickness and degree of exfoliation, can influence electron transport and interfacial contact. Thinner and well-dispersed MXene layers provide more efficient charge-transfer pathways and improved analyte accessibility, although an optimal structural balance is required.

These observations suggest an effective photoinduced charge-transfer process within the EuFeO_3_/ZnO@Ti_3_C_2_T_*x*_ heterostructure. Upon photoexcitation with photon energy equal to or exceeding the band gap (*hν* ≥ *E*_g_), electrons are excited from the valence band (VB) to the conduction band (CB), generating electron–hole pairs. The relatively narrower band gap of EuFeO_3_ enables more efficient absorption in the visible region, while ZnO primarily responds to higher-energy excitation. Driven by favorable band alignment, electrons transfer from the CB of EuFeO_3_ to that of ZnO, while holes migrate in the opposite direction, resulting in effective spatial charge separation. Simultaneously, Ti_3_C_2_T_*x*_ facilitates rapid electron transport and further suppresses recombination. As a result, the synergistic interaction among EuFeO_3_, ZnO, and Ti_3_C_2_T_*x*_ enhances charge-transfer efficiency and carrier mobility within the heterostructure. Such improved charge carrier dynamics are highly beneficial for SERS applications, as they promote stronger electronic coupling and charge-transfer interactions between the substrate and adsorbed analyte molecules, thereby contributing to the chemical enhancement mechanism and leading to enhanced Raman signal intensity.

### 3.6. SERS detection

**Fig. 7:**
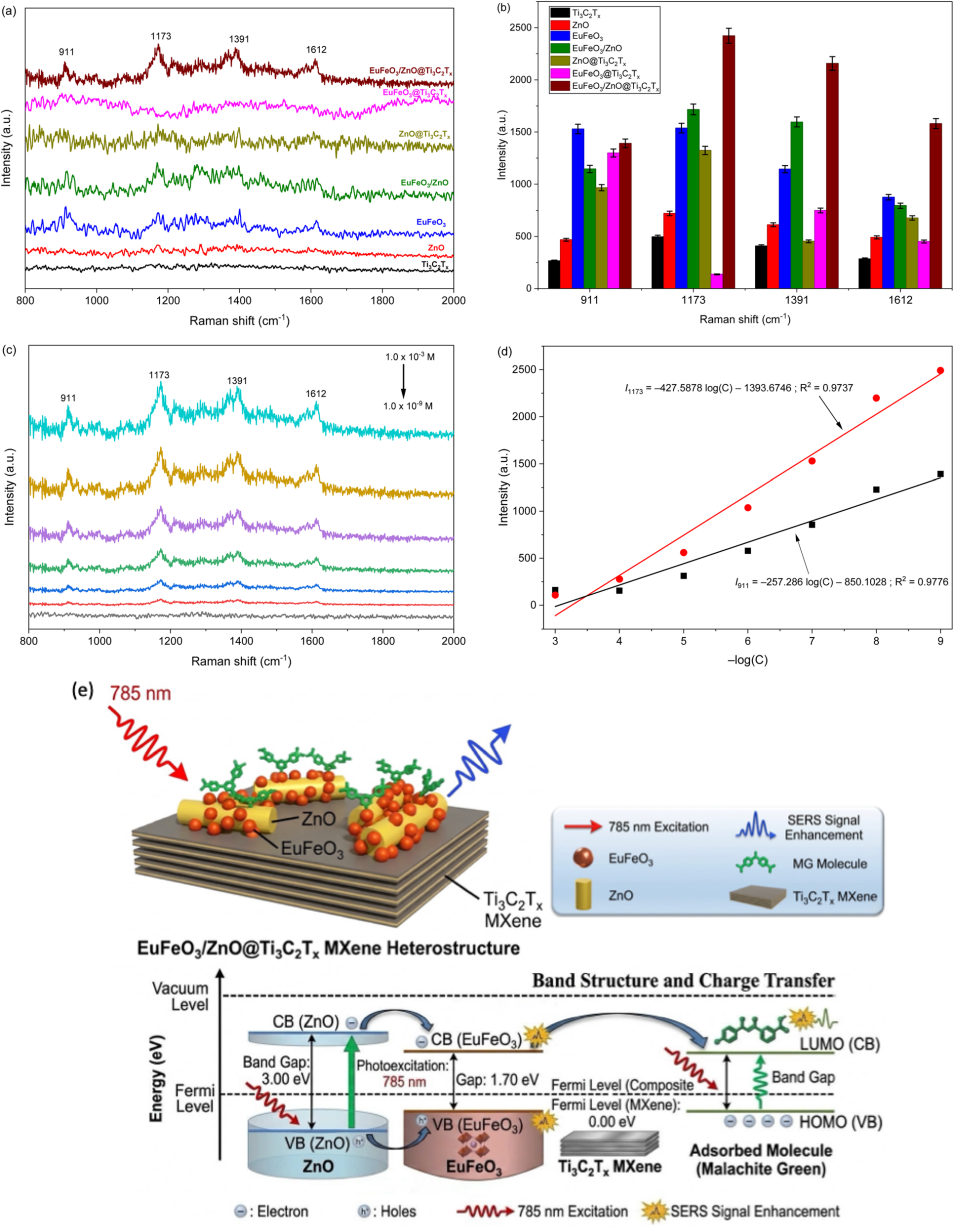
(a) SERS spectra of MG solution on Ti_3_C_2_T_*x*_ MXene, ZnO, EuFeO_3_, and their corresponding binary and ternary composite substrates; (b) comparison of Raman intensities of MG at the characteristic bands of 911, 1174, 1395, and 1615 cm^−1^ recorded on different substrates (*n* = 5); (c) concentration-dependent SERS spectra of MG measured on the EuFeO_3_/ZnO@Ti_3_C_2_T_*x*_ substrate over a concentration range from 10^−9^ to 10^−3^ M; (d) calibration curves showing the linear relationship between MG concentration and Raman intensities at 911 and 1173 cm^−1^; and (e) schematic illustration of the SERS mechanism of EuFeO_3_/ZnO@Ti_3_C_2_T_*x*_ substrate.

Malachite green (MG) solution exhibits several well-defined Raman bands that serve as reliable spectral fingerprints for evaluating SERS performance. As shown in Fig. 7a, the characteristic peaks located at approximately 911 cm^−1^, 1173 cm^−1^, 1391 cm^−1^, and 1612 cm^−1^ are clearly observed. The band at 911 cm^−1^ is attributed to ring breathing and C–N stretching vibrations, while the peaks at 1173 cm^−1^, 1391 cm^−1^, and 1612 cm^−1^ correspond to aromatic C–H in-plane bending, N-phenyl stretching, and combined N–C bonding and aromatic C–C stretching vibrations, respectively. These assignments are consistent with previously reported Raman spectra of MG, confirming the reliability of the spectral identification.^68–73^

Fig. 7b demonstrates a quantitative comparison of the SERS intensities of MG solution at the characteristic Raman bands located at approximately 911, 1174, 1395, and 1615 cm^−1^ on different substrates. Among the pristine materials, Ti_3_C_2_T_*x*_ MXene, ZnO, and EuFeO_3_ exhibit the lowest Raman intensities at all four bands, indicating that none of the individual components alone can provide sufficient enhancement for effective SERS detection. Upon forming binary composites, including EuFeO_3_/ZnO, ZnO@Ti_3_C_2_T_*x*_, and EuFeO_3_@Ti_3_C_2_T_*x*_, the Raman intensities increase noticeably at each characteristic peak. A clear distinction can be observed between the binary systems: the MXene-containing composites (ZnO@Ti_3_C_2_T_*x*_ and EuFeO_3_@Ti_3_C_2_T_*x*_) display stronger Raman signals than the oxide-only EuFeO_3_/ZnO composite, particularly at the bands of 911 and 1174 cm^−1^. This comparison highlights the critical role of Ti_3_C_2_T_*x*_ MXene in promoting signal enhancement, likely by facilitating stronger analyte-substrate interactions and more efficient charge-transfer processes. In contrast, the EuFeO_3_/ZnO@Ti_3_C_2_T_*x*_ ternary composite exhibits the highest Raman intensities across all monitored bands, significantly surpassing not only the pristine substrates but also all binary composites. Importantly, the enhancement provided by the ternary system is consistently observed at all four characteristic peaks, rather than being limited to a single vibrational mode. This uniform amplification across multiple Raman bands indicates a more effective and homogeneous enhancement mechanism. To establish a quantitative basis for comparing the SERS performance of the different substrates, the enhancement factors (EFs) were calculated using the characteristic Raman band at 1173 cm^−1^.

Moreover, the enhancement factor (EF) is an important parameter for quantitatively evaluating the ability of a SERS substrate to amplify Raman signals relative to conventional Raman measurements. In this work, the EF of the substrates was calculated using the standard expression:^74^2
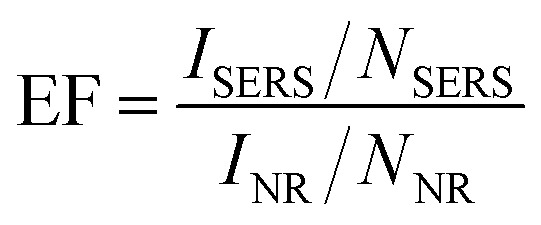
where *I*_SERS_ and *I*_NR_ are the Raman intensities of the same characteristic band measured on the SERS substrate and on a non-enhancing reference substrate (normal Raman), respectively. *N*_SERS_ denotes the number of analyte molecules effectively contributing to the SERS signal within the laser-illuminated area, while *N*_NR_ represents the number of analyte molecules located in the laser sampling volume during normal Raman acquisition. The EF values follow the order: EuFeO_3_/ZnO@Ti_3_C_2_T_*x*_ (7.31 × 10^6^) > EuFeO_3_@Ti_3_C_2_T_*x*_ (6.42 × 10^5^) ≈ ZnO@Ti_3_C_2_T_*x*_ (5.11 × 10^5^) > EuFeO_3_/ZnO (8.14 × 10^4^) > Ti_3_C_2_T_*x*_ MXene ≈ ZnO ≈ EuFeO_3_ (EF < 10^3^), demonstrating that the simultaneous integration of EuFeO_3_, ZnO, and Ti_3_C_2_T_*x*_ MXene produces a synergistic enhancement effect that cannot be achieved by either single or binary systems alone. Although noble-metal-free semiconductor substrates generally produce lower absolute Raman intensities than plasmonic Au- or Ag-based substrates because they lack localized surface plasmon resonance (LSPR), the calculated EF values clearly demonstrate that the ternary heterostructure achieves approximately one order of magnitude higher enhancement than the corresponding binary controls. This substantial improvement can be attributed to the synergistic coupling of the dual Type-II heterojunctions with the highly conductive Ti_3_C_2_T_*x*_ MXene support, which promotes interfacial charge separation, accelerates carrier transport, lowers the charge-transfer energy barrier, and consequently strengthens the chemical enhancement mechanism responsible for SERS signal amplification.

As summarized in Table 1, the specific surface area of the ternary EuFeO_3_/ZnO@Ti_3_C_2_T_*x*_ composite (66.473 m^2^ g^−1^) is lower than that of the binary ZnO@Ti_3_C_2_T_*x*_ system (166.365 m^2^ g^−1^). This decrease in physical surface area is attributed to the successful mass loading of relatively dense, crystalline EuFeO_3_ nanoparticles, which partially fill or block the structural voids and micropores of the layered MXene matrix. Interestingly, despite this reduction in total exposed surface area, the ternary heterostructure delivers a significantly superior SERS enhancement factor (7.31 × 10^6^) compared to its binary counterpart. This clearly demonstrates that physical analyte adsorption capacity is not the limiting or primary factor governing Raman amplification in this system. Instead, the electronic structure plays the dominant role. The integration of EuFeO_3_ introduces a staggered Type-II heterojunction layout that drastically accelerates charge separation, lowers interfacial charge-transfer resistance, and matches the frontier molecular orbitals (HOMO/LUMO) of malachite green. Because chemical SERS enhancement scales directly with the transition dipole moment of the substrate-analyte charge-transfer complex, the highly optimized electronic coupling at the ternary interface overrides the minor loss in physical surface area, yielding superior spectral amplification.

Fig. 7c depicts the SERS spectra of MG solutions acquired on the EuFeO_3_/ZnO@Ti_3_C_2_T_*x*_ substrate at progressively decreasing concentrations ranging from 10^−9^ to 10^−3^ M. As the MG concentration decreases, the intensities of the characteristic Raman bands gradually diminish, while their spectral positions and profiles remain well preserved. Interestingly, even at an ultra-trace concentration of 10^−9^ M, the characteristic Raman features of MG solution remain clearly distinguishable, demonstrating the excellent sensitivity of the engineered ternary substrate. The persistence of well-defined Raman signals at such low concentrations indicates that the substrate provides effective and reproducible signal enhancement rather than sporadic amplification at isolated sites. To further confirm this, SERS spectra collected from 5 randomly selected positions on the ternary substrate yielded a relative standard deviation (RSD) of less than 7% for the core characteristic intensities, firmly verifying the structural homogeneity and spatial reproducibility of the enhancement effect. Across the entire concentration range, the four dominant Raman bands at 911, 1174, 1395, and 1615 cm^−1^, corresponding to the vibrational modes of MG, are consistently observed and retain their relative intensity ratios. This behavior confirms stable analyte–substrate interactions and highlights the suitability of these peaks as reliable spectral markers for MG identification and analysis. In Fig. 7d, calibration curves are constructed by correlating the concentrations of MG solutions over a wide range from 10^−9^ to 10^−3^ M with the Raman intensities of the characteristic bands at 911 and 1173 cm^−1^. A clear linear relationship between Raman intensity and the logarithm of MG concentration is observed for both bands, demonstrating the excellent sensitivity and quantitative reliability of the prepared SERS substrate. The strong linearity over multiple orders of magnitude indicates that the substrate provides consistent signal enhancement without saturation at higher concentrations or excessive signal fluctuation at lower concentrations, confirming its suitability for sensitive and reliable MG detection.

At an MG concentration of 10^−9^ M, the enhancement factor (EF) determined from the characteristic Raman peak at 1173 cm^−1^ is estimated to be on the order of 7.31 × 10^6^, indicating substantial Raman signal amplification even at ultra-low analyte levels. This result further corroborates the high sensitivity of the EuFeO_3_/ZnO@Ti_3_C_2_T_*x*_ substrate and its capability to sustain effective enhancement under trace-level detection conditions. The EF values obtained in the present study, reaching the order of 10^7^, providing strong evidence for the excellent SERS detection capability of the EuFeO_3_/ZnO@Ti_3_C_2_T_*x*_ heterostructure. Unlike noble-metal-based systems where enhancement is dominated by localized surface plasmon resonance, the high EF observed for the EuFeO_3_/ZnO@Ti_3_C_2_T_*x*_ substrate can be predominantly explained by the chemical enhancement mechanism (CEM), which originates from charge-transfer interactions between the adsorbed analyte molecules and the substrate rather than from plasmonic electromagnetic effects. This interpretation is consistent with the noble-metal-free nature of the system and the absence of localized surface plasmon resonance.^75^ In CEM-driven SERS, enhancement arises from photoinduced or ground-state charge transfer between the substrate and the analyte, which alters the electronic structure and polarizability of the molecule and thereby increases its Raman scattering cross-section.^76^ In the present ternary system, the coexistence of EuFeO_3_, ZnO, and Ti_3_C_2_T_*x*_ MXene creates a favorable electronic environment for such charge-transfer processes. The intimate contact between the semiconducting oxides and the conductive MXene scaffold enables efficient electronic coupling at the interface, which is a prerequisite for strong CEM contribution. EuFeO_3_ and ZnO act as charge-transfer mediators, while Ti_3_C_2_T_*x*_ MXene serves as an electron-rich and highly conductive platform.^77^ Under laser excitation, electrons can be transferred between the substrate and the molecular orbitals of MG solution, particularly involving transitions between the substrate electronic states and the highest occupied molecular orbital (HOMO) or lowest unoccupied molecular orbital (LUMO) of MG.^78–80^ To validate these photoinduced charge transfer (PICT) pathways, the band edge potentials of ZnO and EuFeO_3_ were calculated using Mulliken electronegativity equations.*E*_CB_ = *χ* − *E*^e^ − 0.5*E*_g_*E*_VB_ = *E*_CB_ + *E*_g_where *χ* is the absolute electronegativity of the semiconductor (*χ*(ZnO) is approximately 5.79 eV and *χ*(EuFeO_3_) is approximately 5.48 eV), and *E*^e^ is the energy of free electrons on the hydrogen scale (approximately 4.5 eV). For pristine ZnO (*E*_g_ = 3.00 eV), the *E*_CB_ and *E*_VB_ positions were determined to be −0.21 eV and +2.79 eV *versus* NHE, respectively. For EuFeO_3_ (*E*_g_ = 1.70 eV), the *E*_CB_ and *E*_VB_ are located at +0.13 eV and +1.83 eV *versus* NHE, respectively. As illustrated in Fig. 7e, the Fermi level of the metallic Ti_3_C_2_T_*x*_ MXene (*Φ* ≈ −4.65 eV, corresponding to ∼+0.15 eV *vs.* NHE) forms an ideal electron-conduction bridge. Under the excitation of the 785 nm laser (*hν* = 1.58 eV), direct regular band-to-band transitions within the semiconductors are restricted; however, sub-bandgap defect-mediated states and a resonant multi-step interfacial charge transfer are highly favored. Specifically, electrons can be photoexcited from the high-lying valence states of the hybrid heterostructure or the Fermi level of the MXene into the HOMO of the adsorbed MG molecule (−5.44 eV *vs.* vacuum/+0.94 eV *vs.* NHE), or undergo a charge transition from the substrate complex directly to the LUMO level of MG (−3.61 eV *vs.* vacuum/−0.89 eV *vs.* NHE). This robust charge redistribution heavily modulates the molecular polarizability tensor of the analyte, confirming why the chemical enhancement factor is elevated in the ternary system. Such charge-transfer transitions enhance the Raman polarizability tensor of the molecule, leading to selective amplification of vibrational modes that are strongly coupled to the electronic states involved. The dominance of CEM is further supported by the mode-dependent enhancement behavior observed in the SERS spectra.^13^ The pronounced amplification of the characteristic MG bands at 911, 1173, 1395, and 1615 cm^−1^, which are associated with ring breathing, C–N stretching, and aromatic C–C vibrations, suggests strong electronic coupling between the π-conjugated structure of MG and the substrate surface.^81,82^ These vibrational modes are particularly sensitive to changes in molecular polarizability induced by charge transfer, which is a hallmark of CEM-driven enhancement.^83^ In addition, the surface chemistry of Ti_3_C_2_T_*x*_ MXene, characterized by abundant –O and –OH terminations, promotes strong adsorption of MG molecules through electrostatic interactions and hydrogen bonding. This adsorption brings the molecules into close proximity with the active interfaces, increasing the probability of charge transfer and effectively increasing the number of molecules participating in the CEM process.^84–87^ The stable detection of MG down to 10^−9^ M, together with the excellent linearity of the calibration curves, indicates that these charge-transfer interactions are reproducible and uniformly distributed across the substrate surface.

Recent studies have demonstrated that semiconductor-based SERS substrates featuring large specific surface areas, abundant surface defects, and rationally engineered interfacial electronic structures are capable of achieving pronounced Raman signal enhancement, primarily governed by the CEM. A notable example was reported by Xin Jiang *et al.*, who developed a noble-metal-free rGO-TiO_2_-Fe_3_O_4_ nanohybrid in which the cooperative interaction between reduced graphene oxide and semiconductor oxides enabled efficient interfacial charge transfer, resulting in ultra-low detection limits down to the 10^−10^ M level for several probe molecules. In that system, rGO served mainly as an electron acceptor and transport channel, while TiO_2_ and Fe_3_O_4_ provided semiconducting interfaces that promoted charge-transfer transitions between the substrate and adsorbed molecules.^88^ Similarly, Ruma Das and coworkers demonstrated that heteroatom-doped graphene quantum dots (GQDs) can function as effective SERS substrates, where signal enhancement originates predominantly from excited-state charge-transfer processes and modulation of molecular polarizability rather than plasmonic effects. Their results underscored the importance of electronic structure modulation, including heteroatom doping and surface functionalization, in enabling CEM-driven Raman enhancement. However, the enhancement factors reported for GQD-based systems typically remained in the range of 10^3^–10^4^, which can be attributed to the lack of extended conductive pathways for efficient charge transport.^89^ In addition, Dianyu Qi and colleagues reported plasmon-free TiO_2_ photonic microarrays, in which SERS enhancement was ascribed to enhanced light–matter interactions arising from photonic band-gap effects and multiple scattering phenomena. Although this strategy enabled appreciable sensitivity without the use of noble metals, the enhancement mechanism relied strongly on structural optical resonance and precise matching between excitation wavelength and photonic band-gap position, which may impose constraints on flexibility and practical deployment.^90^ By contrast, Yanqiu Zou *et al.* investigated hierarchical CuS microflower architectures, where SERS enhancement was attributed to a combination of charge-transfer interactions and morphology-induced local field effects. Their findings demonstrated that complex semiconductor morphologies with abundant surface features can improve SERS performance, achieving enhancement factors on the order of 10^5^. Nevertheless, such systems often involve mixed contributions from chemical and weak electromagnetic mechanisms, which complicates the mechanistic interpretation of the observed enhancement.^91^

### 3.7. Detection of MG in aquaculture water samples

**Fig. 8:**
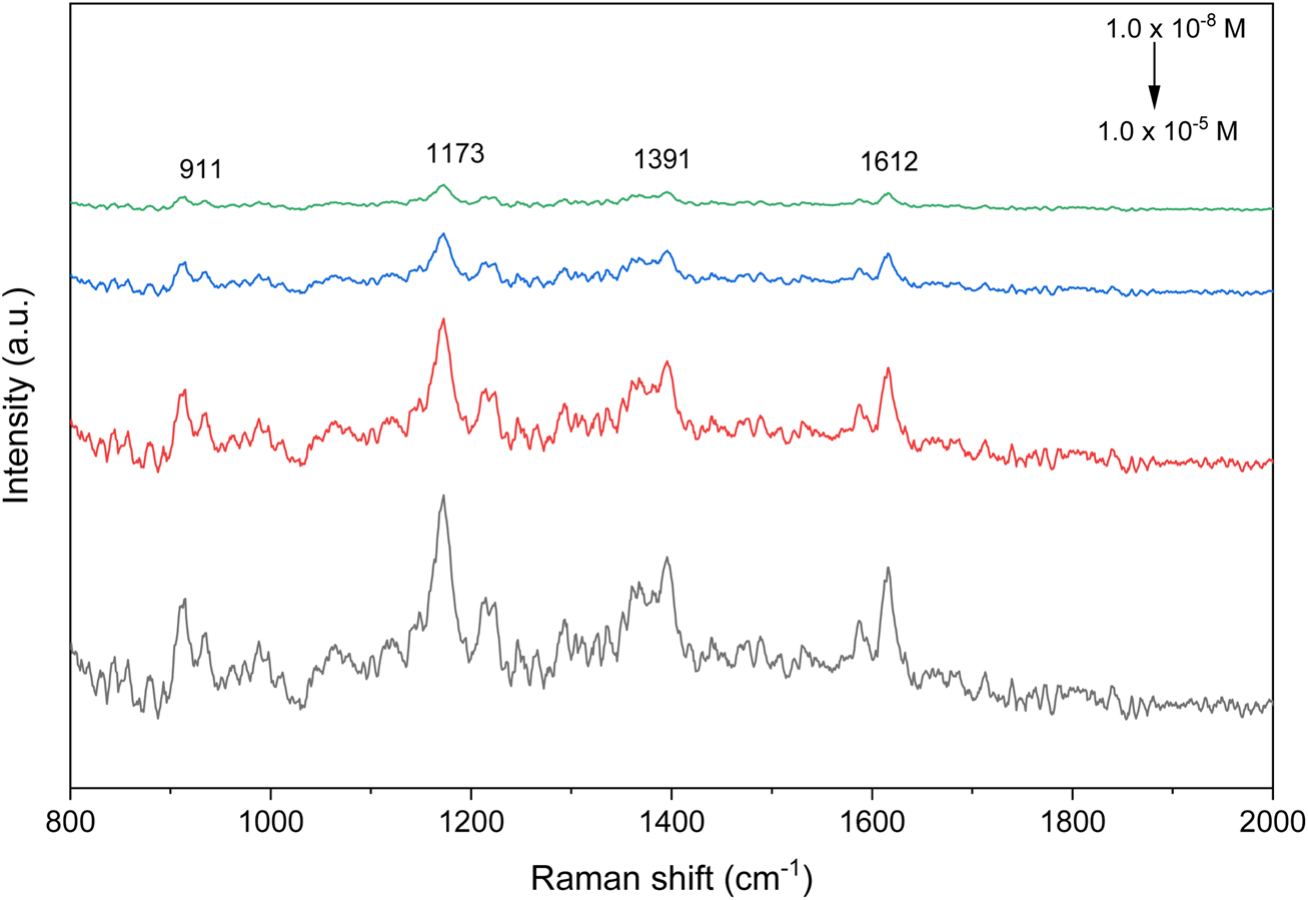
SERS spectra of MG solution on the EuFeO_3_/ZnO@Ti_3_C_2_T_*x*_ substrate in aquaculture water samples spiked with different MG concentrations.

To assess the practical applicability of the developed SERS systems, the EuFeO_3_/ZnO@Ti_3_C_2_T_*x*_ substrate was further applied for the detection of MG in aquaculture water samples. The aquaculture water was collected and used without extensive pretreatment, except for simple membrane filtration to remove suspended particulates, thereby simulating realistic farming conditions. Known concentrations of MG were subsequently spiked into the filtered aquaculture water, and SERS measurements were performed under the same experimental conditions as those used for standard aqueous solutions. As shown in Fig. 8, the characteristic Raman bands of MG located at approximately 911, 1173, 1391, and 1612 cm^−1^ remain clearly identifiable in the aquaculture water matrix, demonstrating the strong signal discrimination capability of the EuFeO_3_/ZnO@Ti_3_C_2_T_*x*_ substrate. Compared with measurements conducted in deionized water, a moderate decrease in Raman intensity is observed, which can be attributed to matrix effects arising from coexisting organic matter and dissolved ions. Nevertheless, the overall spectral profiles and peak positions are well preserved, indicating that the substrate maintains stable and effective SERS activity in complex aquaculture water samples.

As summarized in Table 2, the recoveries of MG in spiked fish pond water samples ranged from 82.70% to 111.80%, indicating acceptable analytical accuracy in the presence of a complex aquatic matrix. The corresponding RSD values ranged from 8.70% to 13.80%, which fall within acceptable limits for SERS-based analysis of real samples, particularly at low and ultra-trace concentration levels. The gradual decrease in recovery and the increase in RSD observed with decreasing MG concentration can be attributed to matrix interference and heterogeneous adsorption effects commonly encountered in chemical-enhancement-dominated SERS measurements. Despite these challenges, the EuFeO_3_/ZnO@Ti_3_C_2_T_*x*_ substrate demonstrates reliable performance for MG detection in fish pond water.

**Table 2:** Determination of MG in aquaculture water samples using the EuFeO_3_/ZnO@Ti_3_C_2_T_*x*_ SERS substrate (*n* = 3)

Sample	Added (M)	Found (M)	Recovery (%)	RSD (%)
Fish pond water	1.0 × 10^−5^	0.89 × 10^−5^	88.6	8.7
	1.0 × 10^−6^	1.12 × 10^−6^	111.8	9.9
	1.0 × 10^−7^	0.84 × 10^−7^	84.3	11.6
	1.0 × 10^−8^	0.83 × 10^−8^	82.7	13.8

## 4. Conclusion

In this work, we have demonstrated the successful development of a noble-metal-free EuFeO_3_/ZnO@Ti_3_C_2_T_*x*_ MXene heterostructure as a high-performance SERS substrate for ultrasensitive detection of malachite green. The rational integration of EuFeO_3_ and ZnO onto a conductive Ti_3_C_2_T_*x*_ scaffold establishes strong interfacial electronic coupling, promotes efficient charge redistribution, and enhances analyte-substrate interactions. As a result, the ternary heterostructure exhibits significantly improved SERS activity compared with pristine and binary counterparts, enabling trace-level detection down to 10^−9^ M with a high enhancement factor of 7.31 × 10^6^. Mechanistic analysis reveals that the observed SERS enhancement is predominantly governed by chemical enhancement, arising from synergistic interfacial charge-transfer processes facilitated by the MXene support rather than plasmonic electromagnetic effects. Importantly, the substrate maintains reliable analytical performance in real aquaculture water samples, delivering acceptable recoveries and reproducibility despite matrix interference, thereby confirming its robustness and practical applicability under realistic environmental conditions. Therefore, this work provides clear insight into the rational design of MXene-based semiconductor heterostructures for chemical-enhancement-dominated SERS sensing and offers a cost-effective and scalable alternative to noble-metal-based substrates. The proposed strategy shows strong potential for practical monitoring of prohibited dyes in aquaculture systems and may be readily extended to the detection of other hazardous contaminants in complex food and environmental matrices. Future work may focus on improving long-term stability and regeneration capability, as well as integrating the MXene-based heterostructure into portable or *in situ* SERS devices to further enhance on-site monitoring applications.

## Author contributions

Thi Thanh Huong Nguyen: conceptualization, investigation, methodology, writing – original draft. Anh Tien Nguyen: methodology, investigation, validation. Tri Danh Giang: formal analysis, visualization, methodology, investigation, writing – original draft. Thi Thuy Pham: supervision, project administration, writing – review & editing. Xuan Vuong Bui: formal analysis, data curation. Thi Lan Huong Nguyen: investigation, visualization. Huu Tri Nguyen: investigation, resources. Ngoc Nhiem Dao: methodology, investigation, data curation, software. Thi Thanh Thuy Le: conceptualization, supervision, funding acquisition, writing – review & editing.

## Conflicts of interest

The author(s) declare that there are no potential conflicts of interest.

## Funding

This research was supported by Saigon University through grant number CSA.2025.110. The author(s) sincerely thank Saigon University for their funding and support of this study.

## Supplementary Material

RA-OLF-D6RA04328C-s001

## Data Availability

Data will be made available on request. Supplementary information (SI) is available. See DOI: https://doi.org/10.1039/d6ra04328c.
