# Supplementary material for: Ultrasensitive SERS detection of trace malachite green using noble-metal-free EuFeO3/ZnO@Ti3C2Tx MXene heterostructures
Source: RSC Adv. 2026 Aug 4;16(39):44648–66. doi: 10.1039/d6ra04328c (PMC13435850; doi:10.1039/d6ra04328c)
Supplement: Supplementary file 1 [file RA-016-D6RA04328C-s001.pdf]

Supplementary Information (SI) for RSC Advances.
This journal is © The Royal Society of Chemistry 2026

# Ultrasensitive SERS detection of trace malachite green using noble-metal-free $EuFeO_3/ZnO@Ti_3C_2T_x$ MXene heterostructures

Thi Thanh Huong Nguyen[a], Anh Tien Nguyen[b], Tri Danh Giang[b], Thi Thuy Pham[c], Xuan Vuong Bui[c], Thi Lan Huong Nguyen[c], Huu Tri Nguyen[c], Ngoc Nhiem Dao[d], Thi Thanh Thuy Le[c,*]

[a] Faculty of Environment and Labour Safety, Ton Duc Thang University, 19 Nguyen Huu Tho Street, Tan Hung Ward, Ho Chi Minh City, 700000, Viet Nam

[b] Faculty of Chemistry, Ho Chi Minh City University of Education, Ho Chi Minh City, 700000, Viet Nam

[c] Faculty of Natural Sciences Education, Sai Gon University, 273 An Duong Vuong Street, Cho Quan Ward, Ho Chi Minh City, 700000, Viet Nam

[d] Institute of Materials Science, Vietnam Academy of Science and Technology, 18 Hoang Quoc Viet Street, Nghia Do Ward, Hanoi, 100000, Viet Nam

* Corresponding author.

E-mail address: lttthuy@sgu.edu.vn (T. T. T. Le)

**SUPPLEMENTARY INFORMATION**

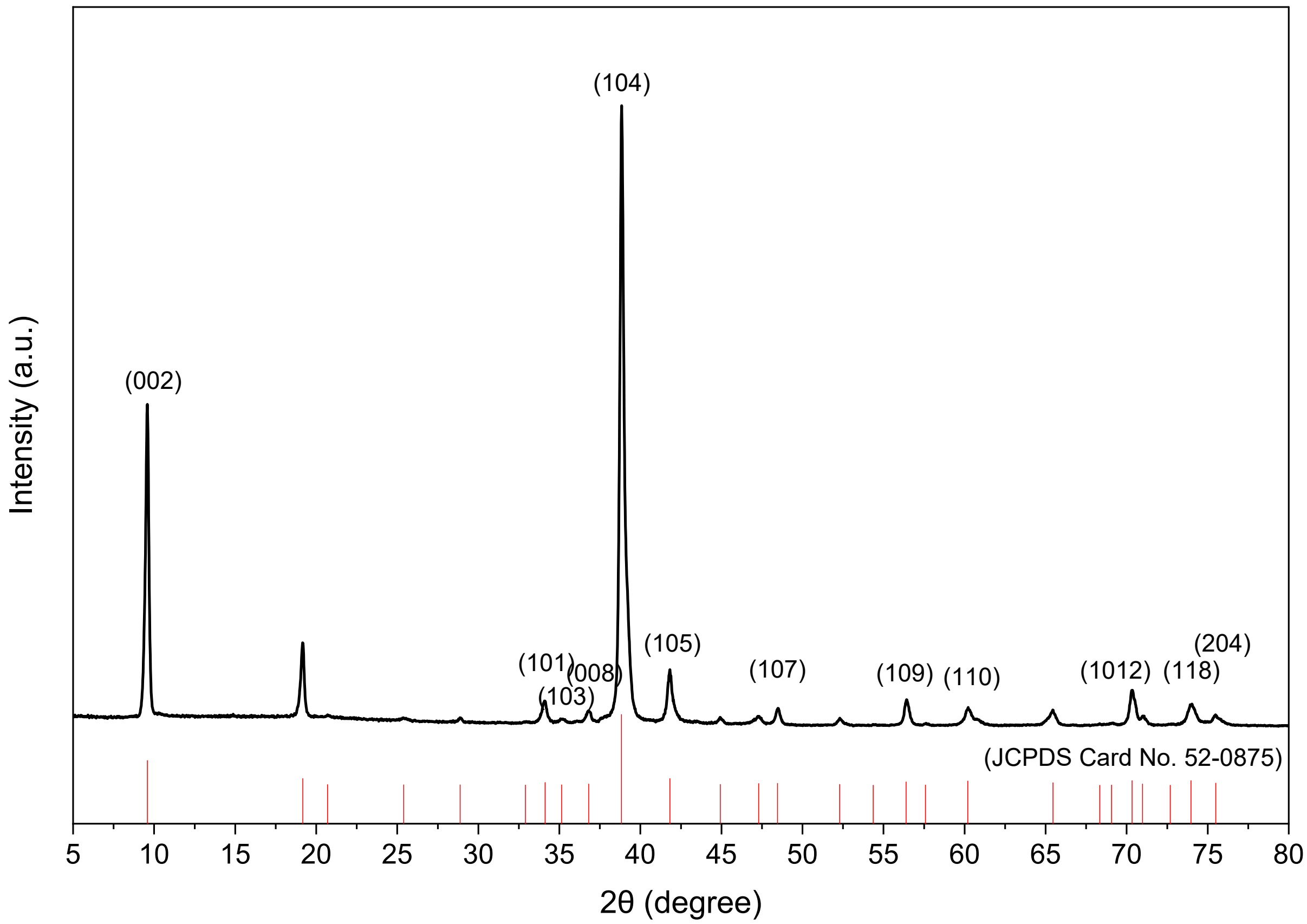


**Fig. S1.** XRD of $Ti_3AlC_2$ MAX
